# Formulation and Performance of NBR/CR-Based High-Damping Rubber Composites for Soundproof Using Orthogonal Test

**DOI:** 10.3390/polym15092208

**Published:** 2023-05-06

**Authors:** Xiankui Zeng, Jinmei Zhu, Gaowei Li, Qing Miao, Mohini Sain, Ranran Jian

**Affiliations:** 1 College of Electromechanical Engineering, Qingdao University of Science and Technology, Qingdao 266061, China; 2 Centre for Biocomposites and Biomaterial Processing, Department of Mechanical and Industrial Engineering, University of Toronto, Toronto, ON M5S 3B3, Canada

**Keywords:** NBR/CR acoustic composites, functional material filler, damping property, sound insulation, orthogonal test

## Abstract

Multiple functional-material-filled nitrile butadiene rubber/chloroprene rubber (NBR/CR) acoustic composites were extensively studied and prepared. According to the orthogonal test table L25 (5^6^), 25 groups of samples were prepared by using a low-temperature one-time rubber mixing process. With tensile strength, average transmission loss, and damping peak as indexes, the influence degree of different factors and levels on the properties of acoustic composites was quantitatively discussed and analyzed. The matrix weight analysis was employed to optimize the material formula of rubber composites, and the corresponding influence weight was given. Results showed that the acoustic composite with blending ratio of 70/30 for NBR/CR matrix had preferable mechanical and acoustic properties; adding mica powder (MP) and montmorillonite (MMT) in matrix contributed to improve all above three indexes owing to their specific lamellar structures; hollow glass beads (HGB) had a positive influence on improving acoustic property due to its hollow microcavities, however, it had a negative impact on damping property because of the smooth spherical surfaces. Accordingly, the optimal formulation was found to be NBR/CR blending ratio of 70/30, MP of 10 phr (per hundred rubber), HGB of 4 phr, and MMT of 10 phr.

## 1. Introduction

Rubber, a highly viscoelastic polymer, is an irreplaceable key material for modern aerospace [1], aviation [2], automotive [3], shock absorbers, and soundproofing systems [4,5]. However, it has a narrow application in some special occasions, such as high-speed train floating floors, ship or aircraft cabin panels and other cutting-edge industrial fields. Unlike general mechanical fields, these fields require materials with large damping factor, excellent mechanical properties, and also to meet the requirements of sound insulation properties. Preparation of rubber composite materials that meet the requirements of multiple performance indexes and can be applied to more sophisticated industrial fields is an urgent problem to be solved.

In recent years, the design of elastomeric nanocomposites with excellent properties has aroused great interest, and numerous researches have demonstrated that the rubber–filler and rubber–rubber interactions are one of the important approaches to compensate for the single property of rubber materials [6,7], including mechanical properties, rheological properties, thermal conductivity, etc. [8,9,10]. Qu et al. [11] reported that nanoclay (NC) and carbon black (CB) filling natural rubber (NR) could effectively improve the mechanical properties, and this improvement was largely dependent on the NC–CB mesh structures, rather than the strain-induced crystallization during the elongation of natural rubber. Tang et al. [8] incorporated the organically modified montmorillonite (OMMT) into chlorobutyl rubber (CIIR) and found that the addition of OMMT improved the tensile strength and hardness, as well as the stress relaxation rate, of CIIR nanocomposites. Several studies have also revealed that the combination of mica powder (MP) with other fillers improved the mechanical properties of composites under tensile and tearing modes, such as MP/wollastonite-filled styrene-butadiene rubber (SBR)/natural rubber (NR) [12] and MP/graphite flakes-filled ethylene propylene diene monomer (EPDM) [13]. In addition, due to the unique structure of hard glass on the outside and inert gas on the inside, hollow glass beads (HGB) are also widely used to fabricate polymer composites for mechanical reinforcement in different applications, such as nitrile and silicone rubbers [14,15].

Generally, for obtaining good sound insulation in occasions, application of sound absorption and isolation materials to attenuate or eliminate sound waves during transmission is an effective way, in addition to controlling the noise sources [16]. Among various materials, rubber exhibits specific viscoelastic and damping properties that have been proven to play an important role in sound insulation and noise reduction, which can convert the kinetic energy generated by the sound waves incident on the material surface into thermal energy [17,18]. Sukontasukkul et al. [19] took advantage of this viscoelastic characteristic of rubber materials, and attached the viscoelastic polymer sheets (VPS) onto the surface of ceramic tiles to improve the damping and sound insulation effect of ceramic tiles. Najib et al. [20] studied the correlation between acoustic and dynamic mechanical properties of natural rubber foam, and proved that the viscoelastic and damping properties of matrix had a significant influence on the acoustic efficiency. In addition, it has also been confirmed that the strong interaction between polymers and fillers with unique structures such as high specific surface area and hollow structure can increase the transmission path of sound waves and improve the sound transmission loss of composites [21,22]. Based on this, Xia et al. [23] fabricated the low-density polyethylene (LDPE) filled by mica with high specific surface area, and their results indicated that the LDPE/mica composites exhibited better sound insulation and mechanical properties compared to the pure LDPE. Wang et al. [24] also pointed out that the introduction of mica contributed to the acoustic friction loss and increased the sound insulation capacity of PVC/mica composites. Zhang et al. [25] reported that the synergistic effect of polyurethane elastomer and hollow glass microspheres improved both damping and sound insulation properties of composites. In our previous work, the sound insulation properties of six kinds of rubber were investigated in detail, and results showed that a combination of nitrile butadiene rubber (NBR) and chloroprene rubber (CR) had excellent damping and sound insulation properties. In addition, fillers such as montmorillonite (MMT), hollow glass beads (HGB), barium sulfate (BaSO_4_), and mica powder (MP) were beneficial to the soundproof properties of rubber composites [26].

In this paper, we tried to investigate the rubber combination and hybrid fillers, making use of the benefits provided by the individual components, aiming to find out their synergistic effect for the acoustic composites by orthogonal test design. The blending ratio of NBR/CR matrix and fillers content of HGB, MMT, and MP were selected as the test factors, while tensile strength, average transmission loss, and damping peak were employed as the test indexes. The influence of four factors on the test indexes were evaluated quantitatively, and the best formula was given by matrix weight analysis.

## 2. Raw Materials and Methodologies

### 2.1. Raw Materials

NBR, brand N41, was supplied by Duokang Industrial Co., Ltd., Shanghai, China (29% acrylonitrile, ρ = 0.96 g/cm^3^, average molecular weight is 700,000). The CR, brand 230, was gained from Shanxiang Industrial Co., Ltd., Shanghai, China (ρ = 1.24 g/cm^3^, average molecular weight is 170,000). MP (1250 mesh) was gained from Lingshou County Huayuan Co., Ltd., Shijiazhuang, China. HGB (2500 mesh) was obtained from Yinuo New Material Co., Ltd., Dongying, China. MMT (2500 mesh) was provided by Lingshou County Yuntao Mineral Products Trading Co., Ltd., Shijiazhuang, China.

### 2.2. Orthogonal Test Design

The orthogonal test is a design method widely used to investigate the multifactor complex system for system design and optimization [27,28]. In the present work, considering comprehensively coupling interaction of the three functional material fillers on the rubber compound system, an orthogonal experiment was designed to obtain the optimal formula of sound insulation materials that met the high damping and excellent mechanical properties requirements. The orthogonal experiment was carried out by adopting four factors and five levels, totaling 25 groups of test, as explained in Table 1. For the detailed process and method of composite materials, the reader can refer to our previous research and work [26].

### 2.3. Characterization

#### 2.3.1. Mechanical Properties

Tensile strength was determined with a TS 2005b tensile testing machine (U-CAN Dynatex Inc., Taiwan, China). The test samples were fabricated into dumbbell style and were stretched at a rate of 50 mm·min^−1^ following the GB/T528-1998 standard. For each composite, the reported values were calculated by taking the average data of 5 samples.

#### 2.3.2. Damping Properties

The damping peak (tan*δ*_max_) was measured under the dynamic mechanical performance test, which was carried out following the GB/T33061.1-2016 on a DMA-150 dynamic mechanical analyzer (NETZSCH GABO Instruments GmbH, Ahlden, Germany). The samples (30 × 6 × 2 mm) were tested in a uniaxial tensile mode with test frequency of 10 Hz, temperature range from −40 to 40 °C, and heating rate of 3 °C min^−1^. Accordingly, the damping property is characterized by Equation (1).

(1) $$\tan\delta =E^{\prime \prime }/E^{\prime }$$

where tan*δ* is the damping factor, *E*″ is the loss modulus, and *E*′ is the storage modulus, respectively.

#### 2.3.3. Sound Insulation Properties

Sound insulation property was characterized by the average transmission loss (TL_avg_), measured by an SW422/SW477 dual-channel impedance tube (BSWA tech., Beijing, China) within fabricated circular samples (2 mm thickness). The diameter of the SW422 tube was 100 mm with the testing frequency range of ≈63–1600 Hz, and the diameter of the SW477 tube was 30 mm with the testing frequency range of ≈1000–6300 Hz. Each sample was tested 5 times, being rotated a certain angle each time. Accordingly, the transmission loss can be calculated using Equation (2).

(2) $$R=10\mathrm{lg}\left(\frac{1}{\tau }\right)$$

where *R* is the sound transmission loss and *τ* is the transmission coefficient, representing the ratio of transmitted sound energy to incident sound energy.

## 3. Test Results and Analysis of Orthogonal Experiment

### 3.1. Orthogonal Test Results

As mentioned above, the tensile strength, tan*δ*_max_, and TL_avg_ were selected as the indicators to evaluate the overall performance of the fabricated acoustic composites. Table 2 illustrates the data of the 25 test groups.

### 3.2. Analysis of Orthogonal Test Results

#### 3.2.1. Range and Variance Analysis

The range analysis method can accurately and quickly determine the primary and secondary relationship of various factors, finding out the preferred combination [29]. As illustrated in Equation (3), *K_ij_* represents the mean value of the test indicators at the same level of each factor, and helps to find out the optimal combination of levels. *R_i_* reflects the change range of the test index when the factor level fluctuates; therefore, the greater the range *R*, the greater the influence of the factor on the test index.

(3) $$R_{i}=\max\left\{K_{ij}\right\}-\min\left\{k_{ij}\right\}\left(i=A,B,C,D;j=1,2,3,4,5\right)$$

The variance analysis is used to determine the influence of control variables on the results and also to verify the credibility of the range analysis [30]. There is a critical value, the so-called *p*-value, which defines the significance of each independent factor. If the *p*-value is larger than 0.05, the effect of the factor is not statistically significant; if the *p*-value is between 0.05 and 0.01, the effect of the factor is statistically significant; and if the *p*-value is less than 0.01, the effect of the factor is statistically highly significant. The calculation process of the variance analysis is shown in Equations (4)–(6).

(4) $$MS_{f}=\frac{SS_{f}}{df}$$

(5) $$MS_{e}=\frac{SS_{e}}{df}$$

(6) $$F=\frac{MS_{f}}{MS_{e}}$$

where *SS_f_* refers to the sum of square of each factor, *SS_e_* refers to the sum of square of error, *df* refers to the degrees of freedom, *MS_f_* refers to the mean square of each factor, *MS_e_* refers to the mean square of error, and *F* refers to the *F*-value. The *p*-value is obtained by looking up the relevant empiric value table based on the calculated *F*-value.

#### 3.2.2. The Mechanical Properties Robustness Analysis

**Figure 1:**
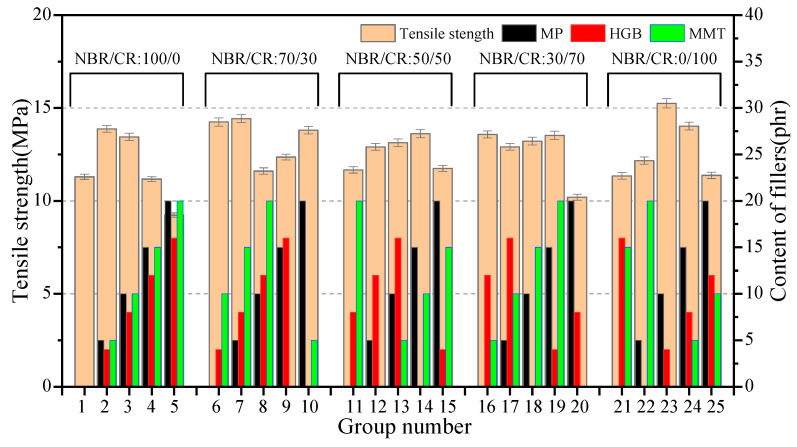
Variation of tensile strength in the orthogonal test.

The tensile strength of samples in the orthogonal test are shown in Figure 1. In the 25 groups of test, the tensile strength of the single CR samples (no. 21–25) was slightly higher than the single NBR samples (no. 1–5). This can be attributed to the fact that CR belongs to self-reinforcing rubber, and the strain-induced molecular chain crystallization during stretching could play a reinforcing role in tensile strength. In addition, from the variations of the no. 1–5 group, it was not hard to find that functional material fillers MP/HGB/MMT could impact the tensile strength. With the increase of filler dosage, the tensile strength appeared to be maximum and then gradually decreased. This inferred that the mechanical properties of the composite were affected by the rubber matrix, as well as the type and amount of filler.

**Figure 2:**
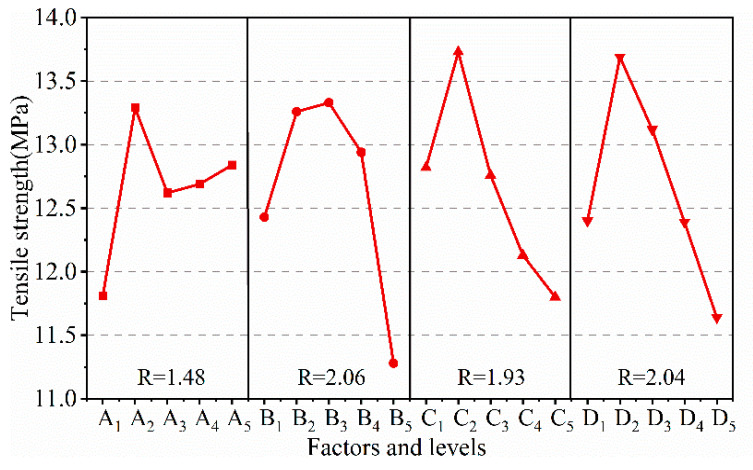
Range analysis for tensile strength of composites.

Figure 2 plots the range analysis results of tensile strength. It can be seen that the R values of factors (A–D) on the tensile strength were 1.48, 2.06, 1.93, 2.04, respectively, so that the impact order of each factor on tensile strength was B > D > C > A. The optimal formulation combination of composite is B_3_D_2_C_2_A_2_ when referring to tensile strength as the evaluation index. It was noteworthy from the curve trend of factor A that blending CR to NBR improved the mechanical properties of NBR, and the sample with NBR/CR blending ratio of 70/30 exhibited the maximum tensile strength, although the NBR/CR blending ration performed the least among these four factors.

The variance analysis results, illustrated in Table 3, showed that the *p*-values of MP and MMT were less than 0.05 while those of NBR/CR ratio and HGB were larger than 0.05, indicating that tensile strength of composites was sensitive to the content of MP and MMT, and HGB and NBR/CR ratio came second, which is consistent with the results of range analysis.

#### 3.2.3. The Sound Insulation Properties Robustness Analysis

**Figure 3:**
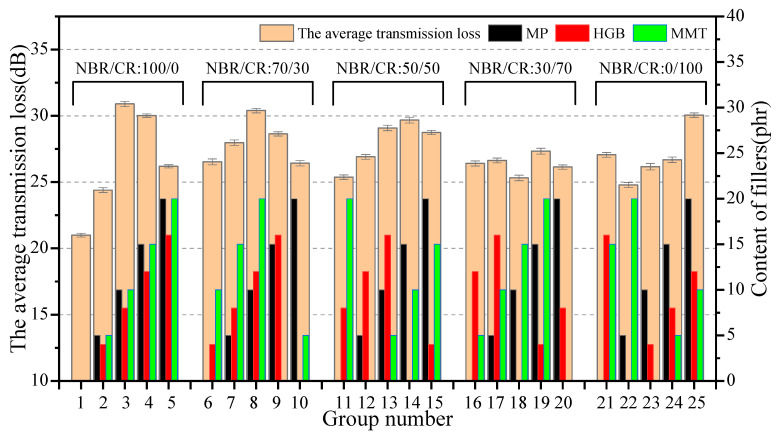
Variation in the average transmission loss of orthogonal test.

Figure 3 illustrates the variation in average transmission loss (TL_avg_) of all specimens from the orthogonal test. Strikingly, the sound transmission loss of the No. 1 group without adding any fillers was significantly lower than that of other groups. This proved that the presence of fillers with specific structures increased the sound transmission loss, resulting in the improvement of sound insulation. Among the same NBR/CR ratio groups (no. 1–5, 6–10, 11–15, 16–20, 21–25), the samples contained no or less than three fillers. No. 1, 10, 11, 18, and 22 had low sound transmission loss, while the samples with multiscale structures by adding all three fillers demonstrated relatively high sound transmission loss. It was further indicated that the synergistic interaction of the lamellar and hollow bead structures of MP/HGB/MMT increased the multiple microwave reflection and absorption, which brought good sound insulation performance.

**Figure 4:**
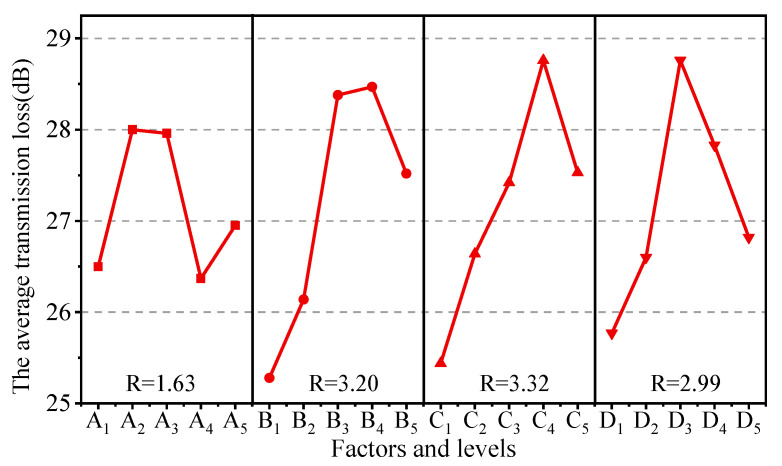
Range curves of the average transmission loss with each factor for composites.

Figure 4 describes the range analysis results of the sound insulation properties. For each factor of fillers, the average transmission loss was quadratically related to the content of fillers, that is, the average transmission loss of the composite system increased and then decreased with the increasing content of fillers. The layered structures with large aspect ratio of MP and MMT and the hollow microcavity structures of HGB enhanced the reflection and scattering of acoustic waves, expanded the distance of acoustic wave propagation, and greatly increased the dissipation rate of acoustic energy in the composites. Therefore, the average transmission loss first increased with the adding of fillers with even dispersion in the rubber matrix. However, with the further adding of fillers, it was easily to induce agglomeration in the case of high amount incorporation, resulting in poor dispersion and uneven distribution of the filler in the polymer matrix, thus affecting the sound insulation properties of composites. The range analysis results present the influence of various factors and levels on the sound insulation following the order C > B > D > A, and the maximum value of four factors is C_4_ = 28.76, B_4_ = 28.47, D_3_ = 28.76, A_2_ = 28.00; i.e., the optimal factor level group combination is C_4_B_4_D_3_A_2_. Specially, according to the R values, sound insulation properties of NBR/CR composites are significantly influenced by MP, MMT, and HGB, yet less affected by the blending ratio of NBR/CR.

The variance analysis results for the average transmission loss were listed in Table 4. It can be found that the *p*-values of MP, HGB, and MMT were all less than the critical value of 0.05, i.e., the fillers were critical in improving the sound insulation properties of the polymer composites, which was consistent with the results of the range analysis. This provides an effective idea to improve the sound transmission loss of rubber composites.

#### 3.2.4. The Damping Properties Robustness Analysis

**Figure 5:**
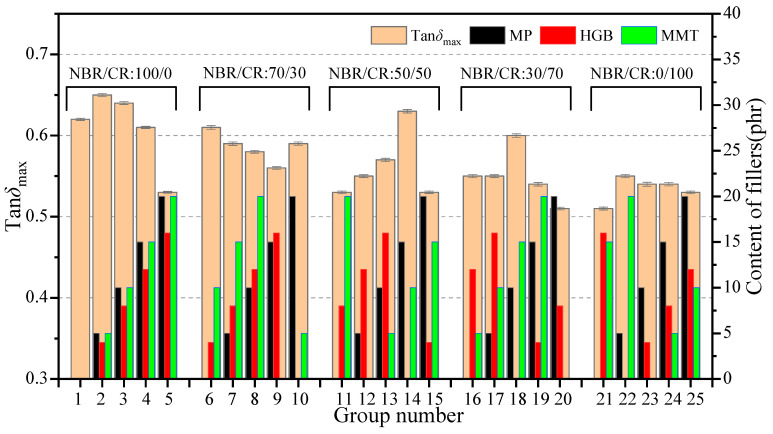
Variation for tan*δ*_max_ of orthogonal test.

Figure 5 plots the variation of damping peak (tan*δ*_max_) of all specimens under dynamic mechanical analysis (DMA). It can be seen from the histogram that the tan*δ*_max_ for the single NBR samples (no. 1–5) was prominently higher than the single CR samples (no. 21–25). As the content of CR increased, tan*δ*_max_ of NBR/CR composites gradually decreased and its values were between that of the two pure rubbers. This can be explained by the fact that the spatial structure of the molecular chain segments became more complex and the motility decreased after blending CR containing polar group (-Cl) to NBR, leading to a decrease in the tan*δ*_max_ of the blended rubbers. In addition, it can be found that the samples without adding HGB exhibited relatively high damping properties among the same NBR/CR ratio group.

**Figure 6:**
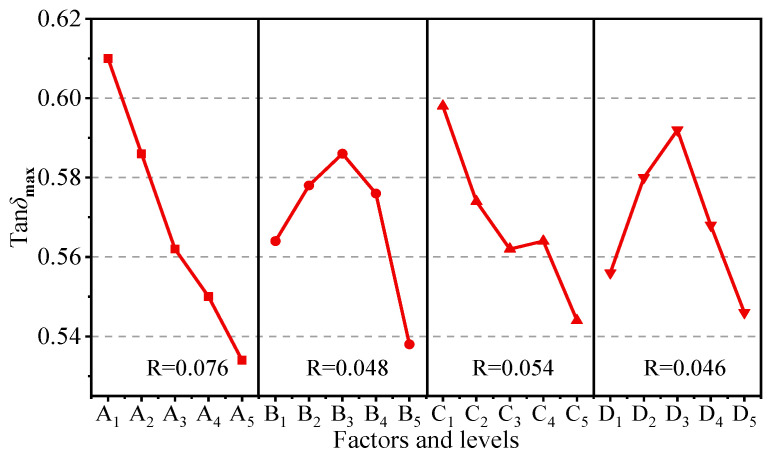
Range curves of tan*δ*_max_ with each factor for composites.

The influence of four factors on the damping properties is shown in Figure 6. From the range analysis results, the range of each factor follows the order of R_A_ > R_C_ > R_B_ > R_D_, that is, the order of the influence for each factor on the damping performance was NBR/CR blending ratio > content of HGB > content of MP > content of MMT, and the optimal combination of factors and levels under this index was A_1_C_1_B_3_D_3_. In addition, it can be concluded that the trends of the MP and MMT curves were similar because they had the similar lamellar structures, that is, tan*δ*_max_ increased first and then decreased with the increasing content. It reached the peak at the content of 10 phr for both MP and MMT. However, HGB had a negative effect on the damping performance. The tan*δ*_max_ decreased undesirably with the increasing content of HGB. One possible explanation is that the friction among molecular chains improved owing to the high specific surface area of lamellar structures of MP and MMT fillers; however, the microsphere structures of HGB filler might increase the risk of molecular chain slippage, which is unfavorable for damping performance.

Variance analysis results in Table 5 indicate the contribution degree for each factor to damping peak (tan*δ*_max_). It is easy to see that the *p*-value for the selected factors were all far less than 0.05, i.e., factors (A–D) all had huge influence on the damping properties of composites.

### 3.3. Weight Matrix Analysis

Given the results of variance analysis and range analysis, three optimal combinations of tensile strength, damping performance, and sound insulation can be obtained, respectively. However, multiobjective optimization still cannot be achieved to obtain a global optimal solution of the three test indexes in the present case.

The analytic hierarchy process (AHP) can quantitatively determine the impact weight of different factors or levels on the test results through matrix calculation to realize quantitative decision analysis [31,32]. Based on the data of the orthogonal experiment, a three-layer structure model was established with the target layer of three indicators, the criterion layer of four factors, and the measurement layer corresponding to the five levels of each factor. Meanwhile, the indicator layer matrix *M*, factor layer matrix *T*, and level layer matrix *S* were defined corresponding to the three-layer structural model [33]. Finally, by calculating *ω = WTS*, the impact weights of each factor and level on tensile strength, TL_avg_, and tan*δ*_max_ were obtained as *ω*_1_, *ω*_2_, and *ω*_3_, respectively. The calculation formulas were as follows:

(7) $$M=\left[\begin{array}{cccc} K_{A1} & & & \\ K_{A2} & & & \\ K_{A3} & & & \\ K_{A4} & & & \\ K_{A5} & & & \\ & K_{B1} & & \\ & K_{B2} & & \\ & K_{B3} & & \\ & K_{B4} & & \\ & K_{B5} & & \\ & & \ddots & \\ & & & K_{D1}\\ & & & K_{D2}\\ & & & K_{D3}\\ & & & K_{D4}\\ & & & K_{D5} \end{array}\right]$$

(8) $$T=\left[\begin{array}{cccc} T_{A} & & & \\ & T_{B} & & \\ & & T_{C} & \\ & & & T_{D} \end{array}\right]$$

(9) $$S=\left[\begin{array}{c} S_{A}\\ S_{B}\\ S_{C}\\ S_{D} \end{array}\right]$$

where $T_{i}=\frac{1}{\sum_{i=1}^{5}k_{ij}}$, $S_{i}=\frac{j}{\sum_{i=1}^{4}R_{j}}$.

**Figure 7:**
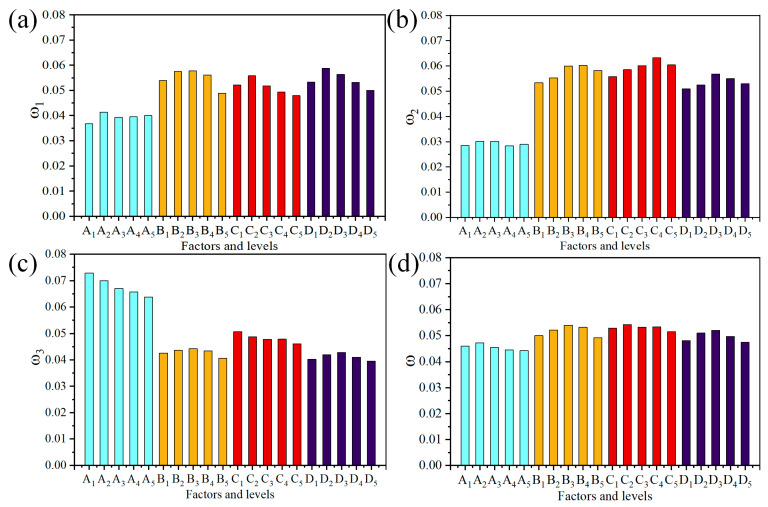
Weight matrix result on indexes: (**a**) impact weights of tensile strength; (**b**) impact weights of TL_avg_; (**c**) impact weights of tan*δ*_max_; (**d**) the average value of the weight of above three indexes.

Owing to different application, the importance of each indicator can be difference. Accordingly, on-demand tailor of functional-material-filled acoustic composites can be achieved by assigning weights to different indicators. The total weight of the orthogonal test index investigated in this paper was the average value of the weight of these three index values, to determine that the influence of mechanical properties, sound insulation properties, and damping properties on the composite materials were consistent. The total weight was calculated by Equation (10) and the results are illustrated in Figure 7.

(10) $$\omega =\frac{\omega_{1}+\omega_{2}+\omega_{3}}{3}$$

As calculated, the influence weights of different factors were A = 0.2274, B = 0.2585, C = 0.2653, and D = 0.2483, respectively. Therefore, the impact order of the four factors was C > B > D > A, revealing that the weight value of factor C was the largest. The maximum weight of test factors at the five levels were A_2_ = 0.0472, B_3_ = 0.0540, C_2_ = 0.0543, and D_3_ = 0.0520, so the optimal factor and level group combination was A_2_B_3_C_2_D_3_.

## 4. Multiscale Synergy of Acoustic Composites

**Figure 8:**
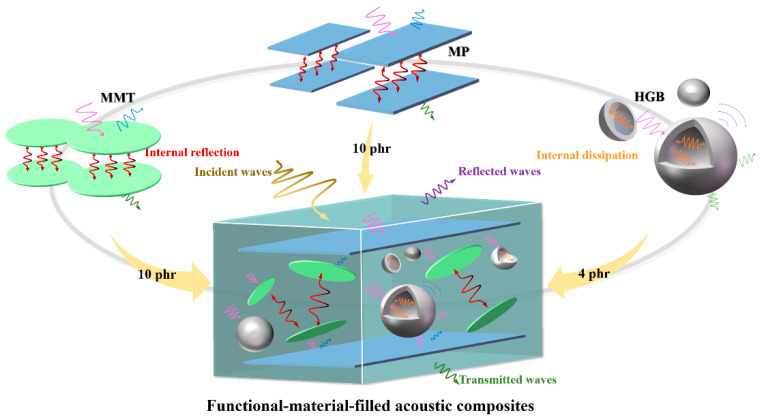
Schematic diagram of sound insulation principle of the prepared high-damping rubber composite with micro-multi-interface and resonant cavity.

Figure 8 plots the sound insulation principle of the prepared high-damping rubber composite with micro-multi-interface and resonant cavity. The matrix used for preparing sound insulation materials was two types of rubbers (nitrile rubber and chloroprene rubber) with high-degree polymerization, the main components of which are copolymers of butadiene and acrylonitrile, as well as polymers of chloroprene. With abundant polar side groups (-Cl and -CN), it can generate strong intermolecular force and friction, so as to obtain good damping performance, which is beneficial to convert external sound energy into heat consumption. In addition, the two rubbers have high density to generate large inertial resistance inside the macromolecule, further causing strong internal friction of the molecular chain for sound attenuation. In addition, in order to further improve the sound insulation performance of rubber composites, different heterogeneous fillers with multiscale structures were added into the rubber matrix to obtain acoustic composites by utilizing a low-temperature one-time rubber-mixing process. Layered structures (MP and MMT) with different microscopic scales allow multiple reflections and scattering of sound waves in the matrix interface array, and gradually consume the energy of sound waves; meanwhile, the unique hollow microcavity structure of HGB allows more reflection and resonance of sound waves, increasing the propagation path of sound waves and the loss of sound energy. Therefore, the coupling between multiscale structure filler and matrix rubber can contribute to sound insulation and noise reduction.

## 5. Conclusions

Types of NBR/CR-based high-damping acoustic composites with micro-multi-interfaces and resonant cavities were designed and investigated. By the tailoring combination of different materials and structures, the unique hollow microsphere structures and inhomogeneous multiscale layered structure were expected to be achieved inside the composites. In this study, 25 groups of different acoustic composites were prepared according to the orthogonal experiment design, and their mechanical properties, acoustic properties, and damping properties were evaluated by range analysis and variance analysis. Finally, the high-damping acoustic composite formulations were screened by weight matrix analysis. The specific conclusions are as follows:

(1) Both the blending ratio of NBR/CR and the content of different fillers have important influence on the mechanical properties, damping properties, and sound insulation properties. The tensile strength of NBR/CR composites increased with adding self-reinforcing CR in the matrix, and the tensile strength of composites was improved by adding various fillers compared to the nonfiller composite; however, the excessive addition of filler might induce the crack propagation of rubber matrix and lead to the rapid reduction of tensile strength.
(2) For sound insulation, both the lamellar structures of mica powder (MP) and montmorillonite (MMT), and the spherical shell surfaces of hollow glass beads (HGB), enhanced the reflection and scattering of acoustic waves, expanded the distance of acoustic wave propagation, and greatly increased the dissipation rate of acoustic energy in the composites. In addition, HGB can also improve the sound absorption ability of the composites through the multiple microwave reflections and resonances in the cell room because of its hollow microcavities.
(3) For the damping properties, the existence of the polar group (-Cl) of CR restricted the motility of molecular chain segments, leading to a decrease of damping, and the lamellar structures in MP and MMT fillers increased the friction among molecular chains owing to the high specific surface area to improve damping; however, the smooth spherical surfaces in HGB filler might increase the risk of molecular chain slippage, which was unfavorable for damping performance.
(4) From the range analysis and variance analysis, it can be concluded that the impact order of each factor on tensile strength was content of MP > content of MMT > content of HGB > NBR/CR blending ratio; the impact order of each factor on sound insulation was content of HGB > content of MP > content of MMT > NBR/CR blending ratio; and the impact order of each factor on damping properties was NBR/CR blending ratio > content of HGB > content of MP > content of MMT.
(5) The weight of the factors (A–D) on each indicator was given quantitatively by the weight matrix analysis method, obtaining the preferred combination of high-damping acoustic composites to be NBR/CR blending ratio of 70/30, MP of 10 phr, HGB of 4 phr, and MMT of 10 phr.

## Figures and Tables

**Table 1:** Experimental factors and levels.

Experimental Factor	Factor	Level				
		1	2	3	4	5
NBR/CR	A	100/0	70/30	50/50	30/70	0/100
MP (phr) *	B	0	5	10	15	20
HGB (phr)	C	0	4	8	12	16
MMT (phr)	D	0	5	10	15	20

* phr, per hundred rubber.

**Table 2:** Orthogonal test design and experimental results.

No.	Factor Level				Test Result		
	NBR/CR	MP	HGB	MMT	Tensile Strength/MPa	tan*δ*_max_	TL_avg_/dB
1	100/0	0	0	0	11.30	0.62	21.00
2	100/0	5	4	5	13.88	0.65	24.40
3	100/0	10	8	10	13.45	0.64	30.90
4	100/0	15	12	15	11.18	0.61	30.02
5	100/0	20	16	20	9.24	0.53	26.20
6	70/30	0	4	10	14.25	0.61	26.53
7	70/30	5	8	15	14.43	0.59	27.98
8	70/30	10	12	20	11.61	0.58	30.40
9	70/30	15	16	0	12.36	0.56	28.64
10	70/30	20	0	5	13.81	0.59	26.43
11	50/50	0	8	20	11.67	0.53	25.38
12	50/50	5	12	0	12.91	0.55	26.91
13	50/50	10	16	5	13.13	0.57	29.09
14	50/50	15	0	10	13.62	0.63	29.68
15	50/50	20	4	15	11.75	0.53	28.75
16	30/70	0	12	5	13.58	0.55	26.41
17	30/70	5	16	10	12.91	0.55	26.64
18	30/70	10	0	15	13.22	0.60	25.32
19	30/70	15	4	20	13.53	0.54	27.34
20	30/70	20	8	0	10.20	0.51	26.14
21	0/100	0	16	15	11.35	0.51	27.06
22	0/100	5	0	20	12.17	0.55	24.79
23	0/100	10	4	0	15.25	0.54	26.17
24	0/100	15	8	5	14.03	0.54	26.69
25	0/100	20	12	10	11.38	0.53	30.06

**Table 3:** Variance analysis for tensile strength of rubber composites.

Factor	*df*	*SS_f_*	*MS_f_*	*F*-Value	*p*-Value
NBR/CR	4	5.775	1.4437	1.94	0.197
MP	4	14.300	3.5749	4.81	0.028
HGB	4	11.032	2.7581	3.71	0.054
MMT	4	12.192	3.0479	4.10	0.043
error	8	5.947	0.7434	-	-

**Table 4:** Variance analysis for the average transmission loss of composites.

Factor	*df*	*SS_f_*	*MS_f_*	*F*-Value	*p*-Value
NBR/CR	4	12.19	3.049	2.25	0.153
MP	4	39.57	9.892	7.29	0.009
HGB	4	29.89	7.472	5.51	0.02
MMT	4	26.80	6.700	4.94	0.027
error	8	10.86	1.357	-	-

**Table 5:** Variance analysis for tan*δ*_max_ of composites.

Factor	*df*	*SS_f_*	*MS_f_*	*F*-Value	*p*-Value
NBR/CR	4	0.018016	0.004504	47.91	0.00001
MP	4	0.007016	0.001754	18.66	0.00041
HGB	4	0.007816	0.001954	20.79	0.00028
MMT	4	0.006736	0.001684	17.91	0.00047
error	8	0.000752	0.000094	-	-

## Data Availability

The data that support the findings of this study are available from the corresponding author upon reasonable request.

## Nomenclature

phr	per hundred rubber
tan*δ*	damping factor
tan*δ*_max_	damping peak
*E*″	loss modulus, N/mm
*E*′	storage modulus, N/mm
*R*	sound transmission loss, dB
TL_avg_	average transmission loss, dB
*τ*	transmission coefficient
*R_i_*	range
*K_ij_*	average of all the results indicator related to the level *j*
*SS_f_*	sum of square of each factor
*SS_e_*	sum of square of error
*df*	degrees of freedom
*MS_f_*	mean square of each factor
*MS_e_*	mean square of error
*F*	*F*-value
*M*	indicator layer matrix
*T*	factor layer matrix
*S*	the level layer matrix
ω	weight matrix
